# Exploratory Study of Soft Drink Intake, Diet, and Body Size Among Employees at a Japanese University Aged 20–39

**DOI:** 10.3390/nu18020292

**Published:** 2026-01-16

**Authors:** Mioko Ito, Kanako Deguchi, Kiyomi Kaito, Risako Yamamoto-Wada, Chihiro Ushiroda, Hiroyuki Naruse, Katsumi Iizuka

**Affiliations:** 1 Department of Clinical Nutrition, Faculty of Medicine, Fujita Health University, Toyoake 470-1192, Japan; 81024002@fujita-hu.ac.jp (M.I.); kanasakuran@gmail.com (K.D.); risako.wada@fujita-hu.ac.jp (R.Y.-W.); chihiro.ushiroda@fujita-hu.ac.jp (C.U.); 2 Health Management Center, Fujita Health University, Toyoake 470-1192, Japan; kkaito@fujita-hu.ac.jp (K.K.); hnaruse@fujita-hu.ac.jp (H.N.)

**Keywords:** sugar-sweetened beverage, SSB, skeletal muscle mass index, SMI, body mass index, BMI

## Abstract

**Background:** Studies outside Japan have linked sugar-sweetened beverage (SSB) intake with weight gain; however, evidence in Japanese adults is scarce, and no study has examined beverage-derived energy in relation to anthropometric indices and handgrip strength. **Methods:** The participants were employees of Fujita Health University aged 20–39 years (*n* = 76; male *n* = 35, average age: 29.97 ± 4.67 years; female *n* = 41, average age: 27.29 ± 4.53 years). Energy from beverage intake was assessed via the Brief Beverage Intake Questionnaire-15, and energy from alcoholic drinks, milk, SSBs, and total beverages was calculated. The associations of energy from different beverages with nutrient intake, BMI, skeletal muscle mass index (SMI), and handgrip strength were analyzed via ordinary least squares (OLS) regression; quantile regression (QR) and the generalized additive model (GAM) were used for sensitivity analyses. **Results:** Increased SSB intake was associated with increased BMI (standardized β = 0.35, 95% CI 0.12–0.58, *p*(OLS) < 0.001; *p*(QR) = 0.23; *p*(GAM) < 0.001) and was nonlinearly associated with increased SMI (standardized β = 0.21, 95% CI 0.043–0.37, *p*(OLS) = 0.02; *p*(QR) = 0.11; *p*(GAM) = 0.02), even after adjustment for total energy intake. Modest milk intake was linked to higher protein intake and a higher SMI without a higher BMI (standardized β = 0.18, 95% CI 0.020–0.35, *p*(OLS) = 0.03; *p*(QR) = 0.39; *p*(GAM) = 0.03). **Conclusions**: A positive association was found between SSB intake and both BMI and SMI and between MILK intake and SMI. Clarification in larger, diverse Japanese populations will be necessary.

## 1. Introduction

Obesity is characterized by multiple cardiovascular risk factors, such as diabetes, dyslipidemia, and hypertension, and in Japan, approximately 30% of men and 20% of women are classified as obese, with the prevalence increasing with age [1,2,3]. Adults in their 20s and 30s are at a life stage when body weight typically begins to increase, making obesity prevention in this age group an important public health issue [2]. In addition to genetic predispositions and age-related hormonal changes, lifestyle factors such as diet, physical activity, and sleep, as well as social and cultural factors such as urbanization and socioeconomic conditions, are thought to contribute to the development of obesity [4].

Among lifestyle factors, excessive intake of sugar-sweetened beverages (SSBs) has been associated with obesity according to numerous epidemiological studies and meta-analyses [5,6,7]. SSBs are a major source of added sugars and are generally characterized by a high glycemic index, which may promote weight gain beyond energy intake through rapid increases in blood glucose levels and the stimulation of intestinal hormones such as insulin and incretins [5]. Moreover, compared with solid foods, beverages tend to induce less satiety and may therefore facilitate excess energy intake [7]. However, the relationships between SSB consumption and body composition parameters (e.g., muscle mass and fat mass), handgrip strength, and energy and nutrient intake have not been fully elucidated. Numerous studies conducted across various countries have investigated the relationship between body mass index (BMI) and the consumption of sugar-sweetened beverages (SSBs). Nevertheless, there is a paucity of evidence concerning the incidence rate of obesity within Japanese populations, despite existing reports that associate SSB consumption with an elevated risk of developing diabetes [8].

Milk and other dairy beverages, on the other hand, are often perceived as health-promoting because they are rich in protein and calcium; however, their fat content may contribute to weight gain [9,10]. The proteins contained in dairy products are efficiently absorbed and may have favorable effects on muscle mass and physical function, but how the consumption of milk-containing beverages affects body composition, handgrip strength, and energy and nutrient intake among Japanese adults is unclear.

In Japan, detailed data on the consumption of beverages such as SSBs, milk beverages, and alcoholic beverages are scarce, particularly for adults in their 20s and 30 s, and the associations of beverage intake with energy and nutrient intake and anthropometric indices are poorly understood. Because people in their 20s and 30 s often experience substantial lifestyle changes, eating and drinking behaviors during this period may influence the future risk of obesity and lifestyle-related diseases [11]. Clarifying beverage consumption patterns and their relationships with health indicators in this age group is therefore important for developing nutritional guidance aimed at preventing obesity.

Therefore, the present study aimed to investigate the associations of energy intake from beverages assessed via the Beverage Intake Questionnaire (BEVQ-15) [12,13], including total beverages, alcoholic beverages, milk beverages, and sugar-sweetened beverages, with usual total energy and macronutrient intake (carbohydrate, fat, and protein), anthropometric indicators (BMI, body fat percentage, skeletal muscle mass index), and handgrip strength among Japanese men and women in their 20s and 30s.

## 2. Materials and Methods

### 2.1. Study Design

This cross-sectional observational study compared the associations between energy from beverage intake and nutrient intake, body size and grip strength in young Japanese adults aged 20–39 years. The participants were employees of Fujita Health University Hospital (*n* = 76; male *n* = 35; female *n* = 41). Consequently, we applied the criteria listed below. The inclusion criteria were as follows: individuals (employees) aged 20–39 years at the time of consent who provided written consent to participate after being informed and demonstrating comprehension. The exclusion criterion also included other staff members deemed inappropriate by the principal investigator or coinvestigators; however, there were no such individuals. Recruitment was conducted on campus in October 2023, aiming for a total of 100 male and female participants. The study was implemented from November 2024 to January 2025. The reason for conducting the study with a small number of participants was that it was positioned as a pilot study from the outset. Therefore, instead of setting a specific number of cases, we set the target at 100 participants, which is the number we are able to recruit.

Individuals who were deemed unsuitable for participation in this study by a physician were excluded. The study was conducted in accordance with the principles of the Declaration of Helsinki and was approved by the Research Ethics Committee of Fujita Health University (approval number HM23-209, approval date: 20 September 2023; approval number HM24-439, approval date 27 January 2025; approval number HM25-451, approval date 9 December 2025). All patients provided written informed consent before inclusion in this study.

### 2.2. Brief-Type Self-Administered Diet History Questionnaire (BDHQ)

The brief-type self-administered diet history questionnaire (BDHQ) is used in Japan to estimate baseline dietary patterns and control for habitual intake [14,15].

### 2.3. Brief Beverage Intake Questionnaire-15 (BEVQ-15)

Usual beverage intake over the past month was assessed with the Brief Beverage Intake Questionnaire (BEVQ-15), a validated 15-item, self-administered tool [12,13]. The participants reported the frequency and usual portion size for each beverage category (e.g., water, sugar-sweetened beverages, milk, fruit juice, and alcoholic drinks), from which the daily volume (mL/day) and energy intake (kcal/day) for each category and for total beverages were calculated. In this analysis, the numbers for energy intake were used.

In the BEVQ-15 questionnaire, the type of beverage includes water or unsweetened sparkling water; 100% fruit juice; sweetened juice beverage/drink; whole milk, reduced milk 2%; purple cap, or chocolate milk; low fat 1%, fat free/skim milk, buttermilk or soy milk; nut milk (almond, cashew, coconut) “O flavor, or plain O unsweetened”; soft drinks, regular; energy & sports drinks, regular (red bull, gatorade, powerade); diet or artificially sweetened soft, drinks, energy & sports drinks, sports drinks, sports drinks, sports drinks, sports drinks; sweet tea (with sugar); tea or coffee, black (no creamer or milk); tea or coffee (w/milk &/or creamer); wine (red or white); hard liquor; beer, wines, wine coolers, nonalcoholic or light beer; others.

From these, each calorie was calculated as follows:

(1) The total SSB caloric content was calculated from sweetened juice drinks, regular soft drinks, sweet tea, and energy drinks (coffee/tea was also included if consumed with sugar and/or a sweetened creamer).
(2) Total milk calories were calculated from whole milk, reduced fat milk, chocolate milk, low fat, fat free/skim, buttermilk or soy milk, and nut milk.
(3) The total alcohol caloric content was calculated from wine, hard liquor, beer, eggs, wine coolers, and nonalcoholic or light beer.
(4) For Nut Milks, if Flavored, original, or plain is selected, 9.8 is used as the value for “avg kcal.” If unsweetened, 4.2 is used as the value for “avg kcal.” For Tea or Coffee categories, SUM is used for those preferences selected, and the value for “avg kcal” is used.

### 2.4. Measurement of Body Size and Grip Strength

Body weight (BW), BF%, and skeletal muscle mass were measured with an InBody Dial H20N weight analyzer (Inbody Japan Inc., Tokyo, Japan). The skeletal muscle mass index (SMI) is a measure of skeletal muscle mass weight and is typically calculated by dividing muscle mass by height squared [16]. The grip strength was calculated as the average strength of both hands. Grip strength was measured with a TOEI LIGHT grip strength tester ST3 T1781 (Toei Light Inc., Souka, Saitama, Japan).

### 2.5. Statistical Analysis

In this study, analyses were conducted via R (R version 4.5.1, 13 June 2025; R Foundation for Statistical Computing, R Core Team, Vienna, Austria [17]). For data processing, we used dplyr (v1.1.x), tidyr (v1.3.x), stringi (v1.8.x), tibble (v3.2.x), purrr (v1.0.x), and readr (v2.1.x). For regression analyses, we used base R’s lm (1.7-2); for quantile regression, we used the quantreg package (v5.97–5.98); and for generalized additive models (GAMs), we used the mgcv package (v1.9.x). We used broom (v1.0.x) to format the results from the models, and ggplot2 (v3.5.x) was used for visualization.

#### 2.5.1. Outcome Variables and Explanatory Variables

The outcomes consisted of the following indicators: nutrients (energy, carbohydrate, protein, and fat) and body size (body mass index (BMI), whole-body skeletal muscle mass index (SMI), body fat (BF), and grip strength). The corresponding variable names were automatically detected from the column names in the data and used.

The exposure variables representing beverage intake were as follows:

Drink (Alc): alcohol beverages
Drink (milk): milk beverages
Drink (sugar): SSB
Drink (total): total beverage intake

#### 2.5.2. Adjustment of the Models

For each combination of outcome and beverage intake, the following two models were estimated:

Model 1: Age + sex
Model 2: Age + sex + total energy intake

Missing values were handled by a complete-case analysis, and only rows with all the required variables were used for the analysis.

#### 2.5.3. Linear Regression (OLS)

OLS models were estimated via the following equation:

Y = β_0_ + β_1_X + β_2_Age + β_3_Sex (+β_4_Energy)

On the basis of the standard deviations of exposure X and outcome Y, standardized regression coefficients (standardized β) were calculated from β_1_. The standard error and 95% confidence interval for standardized β were also derived.

#### 2.5.4. Quantile Regression (QR, τ = 0.5)

To evaluate associations at the median level, quantile regression with τ = 0.5 (median) was conducted using the same adjustment factors. Standard errors were estimated via the nid method, and *p* values were calculated.

#### 2.5.5. Generalized Additive Model (GAM)

To examine the possibility of nonlinear relationships, we constructed GAMs for each outcome–predictor combination via the mgcv package in R. A thin plate regression spline was applied to beverage intake X, with age and sex included as linear terms for adjustment, and models with fewer than 10 valid observations were excluded. For each smoothing term s(X), the effective degrees of freedom (EDF) and *p* value were obtained; the results were summarized in a comma-separated values file, and predictors with *p* < 0.05 and EDF ≥ 2 were considered to have statistically significant nonlinear associations. For these models, age was fixed at the median value and sex at a representative category, and predicted values and 95% confidence intervals across the observed range of X were calculated and visualized as partial effect curves.

#### 2.5.6. Integration and Visualization of the Results

The OLS, quantile regression, and GAM results were integrated and organized into tables, and a summary table comparing the standardized β values of the age + sex model and the age + sex + energy model was prepared. Additionally, using the standardized β and 95% CI from OLS, a forest plot for each outcome was created.

All tests were two-sided, and the significance level was set at 5%.

## 3. Results

### 3.1. Baseline Characteristics of the Participants

When baseline characteristics were compared between men and women, women were significantly younger than men were (29.97 ± 4.67 vs. 27.29 ± 4.53 years, *p* = 0.011). The participants ranged in age from 23 to 39 years. No significant sex difference in BMI was observed (*p* = 0.084). In contrast, women had a greater body fat percentage, whereas men had a greater SMI and grip strength; all of these differences were statistically significant, with large effect sizes (for %BF, *p* = 0.00, Cohen’s d = −1.52; for SMI, *p* = 0.00, Cohen’s d = 2.056 (95% CI 1.496–2.616); and for grip strength, *p* = 0.00, Cohen’s d = 2.059 (95% CI 1.499–2.619)) (Table 1).

**Figure 1:**
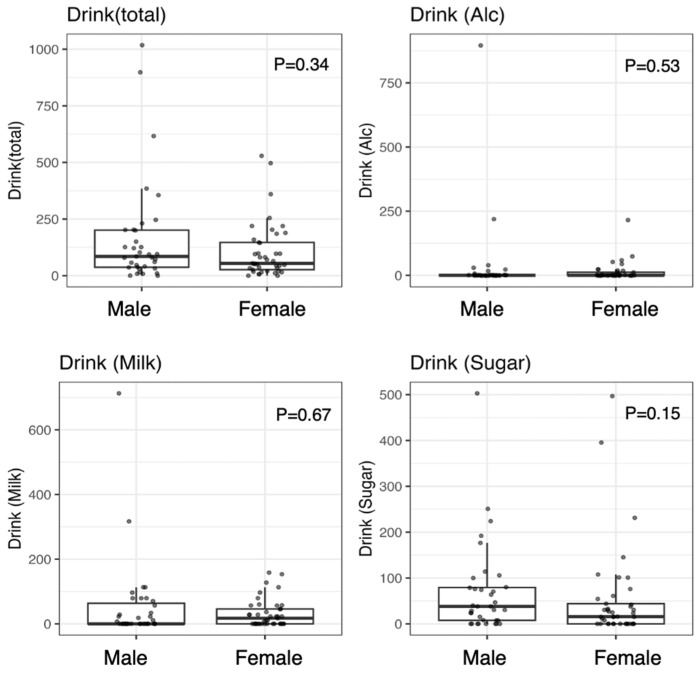
Amount of Daily Beverage Intake among the Participants. Group differences were examined via the Mann–Whitney U test. *p* < 0.05 was considered significant.

In terms of dietary intake, total energy, protein, carbohydrate, and fat intake was significantly greater in men than in women (*p* < 0.01). In contrast, no sex differences in beverage intake were observed; drink (total), drink (Alc), drink (milk), and drink (sugar) did not differ significantly between men and women (all *p* > 0.10) (Figure 1).

### 3.2. The Correlations Between Beverage-Derived Energy and Total Beverage Intake (Drink (Total)), Sugar-Sweetened Beverages, Milk-Containing Beverages, or Alcoholic Beverages

**Figure 2:**
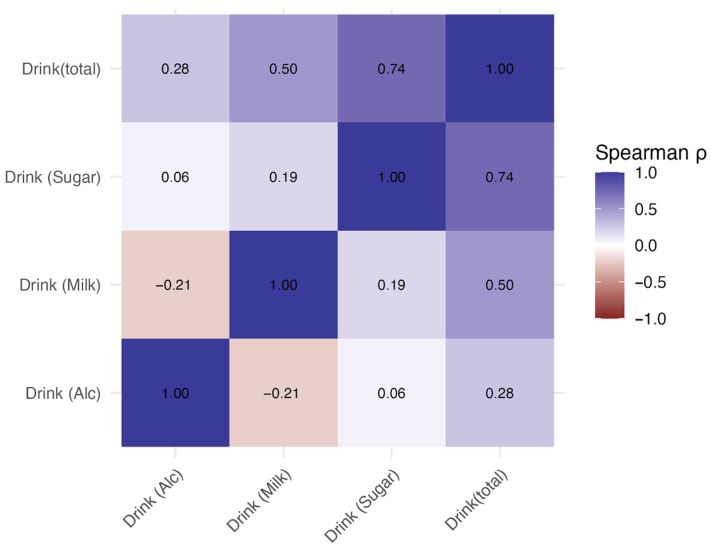
Spearman correlation between beverage intake (total, sugar, milk, and alcohol). Heatmap showing Spearman’s rank correlation coefficients (ρ) between total, sugar, milk, and alcohol beverage intake. The colors indicate the strength and direction of the correlation (purple, positive; red, negative; white, no correlation).

With respect to beverage-derived energy, total beverage intake (drink (total)) was moderately to strongly positively correlated with the consumption of sugar-sweetened beverages (drink (sugar); ρ = 0.74, 95% CI 0.57–0.86, *p* < 0.001) and milk-containing beverages (drink (milk); ρ = 0.50, 95% CI 0.29–0.68, *p* < 0.001). In contrast, alcohol beverage intake (drink (Alc)) showed only weak correlations with the other beverage categories, and none of these correlations reached statistical significance (e.g., drink (Alc) vs. drink (Milk): ρ = −0.21, *p* = 0.063) (Figure 2 and Table 2).

### 3.3. Associations of Beverage Intake with Energy and Nutrient Intake

**Figure 3:**
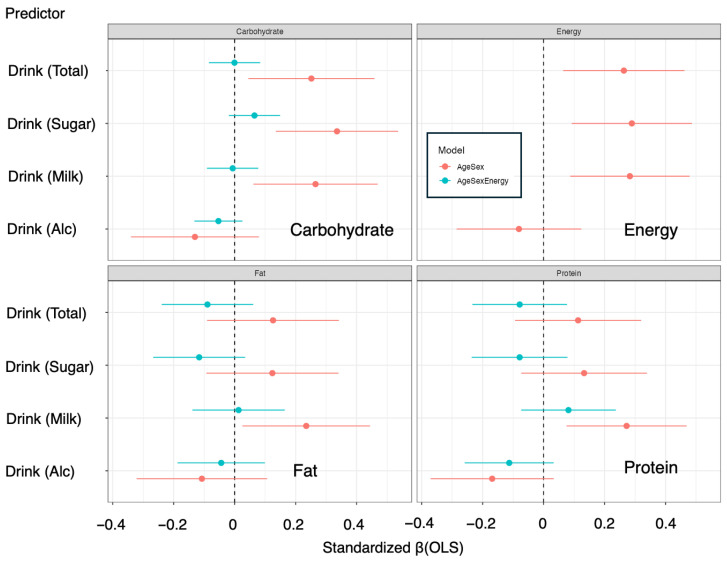
Comparison of standardized β (OLS) values for each nutrient across beverage types. The horizontal axis represents standardized β (OLS), and the vertical axis represents beverage types. Red and blue indicate adjusted Model 1: Age + Sex and Model 2: Age + Sex + Energy, respectively. Standardized regression coefficients (standardized β) were obtained by multiplying the unstandardized coefficient β_1_ by the ratio of the standard deviations of the exposure and outcome, that is, β* = (SD_X/SD_Y) × β_1_, where SD_X and SD_Y denote the sample standard deviations of exposure X and outcome Y, respectively.

To examine the associations of beverage intake with energy and nutrient intake, ordinary least squares (OLS) regression was used as the main analytical approach, and sensitivity analyses were conducted via quantile regression (to account for a skewed distribution) and generalized additive models (GAMs; to explore potential nonlinear relationships). Standardized β coefficients were summarized and compared across beverage categories and outcomes via forest plots (Figure 3).

For total beverage intake, the age- and sex-adjusted models revealed significant positive associations with total energy and carbohydrate intake (energy: β = 0.263 [0.064–0.462], *p* = 0.01; carbohydrate: β = 0.252 [0.045–0.458], *p* = 0.02) (Figure 3 and Table 3). In sensitivity analyses, both quantile regression and the GAM indicated significant effects for these outcomes (Energy: *p*(QR) = 0.01, *p*(GAM) = 0.01; Carbohydrate: *p*(QR) = 0.01, *p*(GAM) = 0.02), supporting the robustness of the associations (Figure 3 and Table 3). However, after additional adjustment for total energy intake (age + sex + energy model), all standardized β values fell below 0.2, and no significant associations were observed in OLS, quantile regression, or GAM, indicating that the relationships were largely attenuated when overall energy intake was considered (Figure 3 and Table 3).

Sugar-sweetened beverages were positively associated with total energy and carbohydrate intake in the age- and sex-adjusted model (energy: β = 0.289 [0.092–0.487], *p* = 0.01; carbohydrate: β = 0.336 [0.135–0.536], *p* = 0.00). These associations were supported by sensitivity analyses via both quantile regression and the GAM (energy: *p*(QR) = 0.00, *p*(GAM) = 0.01; carbohydrate: *p*(QR) = 0.01, *p*(GAM) = 0.00) (Figure 3 and Table 3). However, after additional adjustment for total energy intake, the standardized β coefficients for all nutrient outcomes were less than 0.2, and no significant associations remained in the OLS, quantile regression, or GAM (Figure 3 and Table 3). The associations of sugar-sweetened beverages with fat and protein intake were small (standardized β < 0.2) and nonsignificant in all the models (Figure 3 and Table 3).

**Figure 4:**
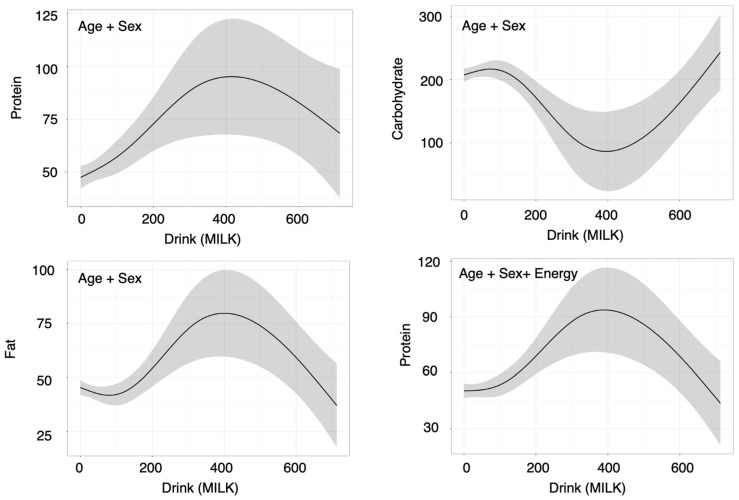
Nonlinear associations between Drink (Milk) and the outcome (protein, carbohydrate, fat) identified by generalized additive models (Model 1: age + sex; Model 2: age + sex + energy) (*p* < 0.05 and EDF ≥ 2). Shadow indicates 95%CI. Predictors with *p* < 0.05 and EDF ≥ 2 were considered to show statistically significant nonlinear associations, and only these associations are depicted as curves in the figures.

In contrast, milk intake showed consistent positive associations with multiple nutrients in the age- and sex-adjusted model. Specifically, milk intake was positively associated with total energy, carbohydrate, fat, and protein contents (energy: β = 0.283 [0.087–0.479], *p* = 0.01; carbohydrate: β = 0.265 [0.061–0.469], *p* = 0.01; fat: β = 0.235 [0.025–0.444], *p* = 0.03; protein: β = 0.272 [0.075–0.469], *p* = 0.01) (Figure 3 and Table 3). In the sensitivity analyses, QR did not consistently reach significance for all the nutrients (energy: *p*(QR) = 0.33; carbohydrate: *p*(QR) = 0.00; fat: *p*(QR) = 0.53; protein: *p*(QR) = 0.69), whereas the GAM demonstrated significant nonlinear effects for each outcome (energy: *p*(GAM) = 0.01; carbohydrate: *p*(GAM) = 0.01; fat: *p*(GAM) = 0.05; protein: *p*(GAM) = 0.01), supporting the robustness of the associations and suggesting potential nonlinearity (Figure 3 and Table 3). After additional adjustment for total energy intake, all standardized β coefficients fell below 0.2, and the associations were no longer significant in the OLS (Figure 3 and Table 3). Nonetheless, the GAM continued to show significant nonlinear effects for all outcomes (all *p*(GAM) = 0.00), indicating that associations between milk intake and nutrient intake may persist within specific intake ranges even after accounting for total energy (Figure 4 and Table 3). The partial effect plots from the GAMs (Figure 4) revealed distinct nonlinear patterns for the relationships between milk intake and macronutrient intake: for protein and fat, the curves suggested steep increases in the outcomes at low-to-moderate levels of milk intake followed by a decrease at higher intakes, whereas for carbohydrate intake, the pattern indicated an initial decrease at low-to-moderate intakes followed by an increase at higher intakes.

Alcohol intake was not significantly associated with energy or any of the macronutrients in either the age- and sex-adjusted or the age-, sex-, and energy-adjusted models; all standardized β coefficients were less than 0.2, and no significant effects were detected via OLS, quantile regression, or GAM (Figure 3 and Table 3).

### 3.4. Associations of Beverage Intake with Body Size

**Figure 5:**
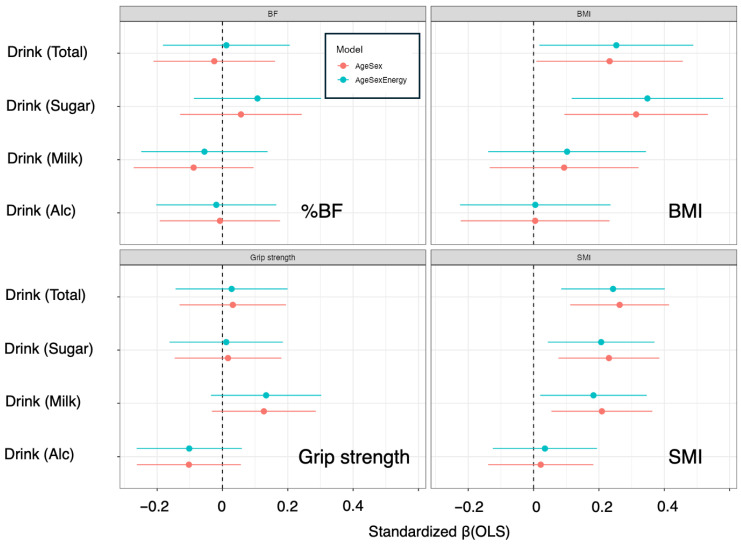
Standardized β (OLS) values for beverages and each nutrient. The horizontal axis represents standardized β (OLS), and the vertical axis represents beverages. Red and blue indicate adjusted Model 1: Age + Sex and Model 2: Age + Sex + Energy, respectively. Abbreviations: %BF, body fat percentage; BMI, body mass index; SMI, skeletal muscle mass index.

The associations between beverage intake and body composition indices were subsequently examined. Total beverage intake was positively associated with both BMI and the SMI in the age- and sex-adjusted model (BMI: β = 0.233 [0.008–0.457], *p*(OLS) = 0.05; SMI: β = 0.263 [0.112–0.415], *p*(OLS) = 0.00) (Table 4, Figure 5). In the sensitivity analyses, no significant associations with BMI were detected by quantile regression or the GAM (*p*(QR) = 0.71, *p*(GAM) = 0.09), whereas both methods supported a significant association with the SMI and indicated nonlinear effects (*p*(QR) = 0.04, *p*(GAM) = 0.00) (Table 4, Figure 5). In the age-, sex-, and energy-adjusted model, total beverage intake remained positively associated with BMI and SMI (BMI: β = 0.253 [0.018–0.489], *p*(OLS) = 0.04; SMI: β = 0.243 [0.085–0.402], *p*(OLS) = 0.00) (Table 4, Figure 5). For BMI, quantile regression and the GAM did not show significant associations in this fully adjusted model (*p*(QR) = 0.71, *p*(GAM) = 0.08), whereas for SMI, the significant nonlinear association persisted (*p*(QR) = 0.03, *p*(GAM) = 0.00) (Table 4, Figure 5). In contrast, no significant associations were observed between total beverage intake and BF or grip strength in any model, and all standardized β coefficients for these outcomes were less than 0.2. Given that beverage intake exhibited a highly skewed distribution, with a substantial proportion of participants reporting no intake, the distribution likely became zero-inflated. Under such conditions, OLS and GAM tend to detect associations driven by the upper end of the intake range, whereas quantile regression, which focuses on the median, may fail to identify an effect. This imbalance may explain why OLS and GAM detected significant associations, whereas quantile regression did not.

**Figure 6:**
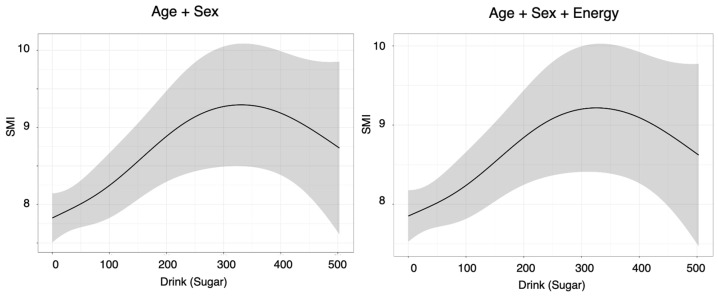
Nonlinear associations between Drink (Sugar) and the outcome (SMI) identified by generalized additive models (model 1: age + sex; model 2: age + sex + energy) (*p* < 0.05 and EDF ≥ 2).) shadow indicate 95%CI. Predictors with *p* < 0.05 and EDF ≥ 2 were considered to show statistically significant nonlinear associations, and only these associations are depicted as curves in the figures.

Sugar-sweetened beverages were positively associated with BMI and SMI in the age- and sex-adjusted model (BMI: β = 0.314 [0.094–0.533], *p*(OLS) = 0.01; SMI: β = 0.231 [0.076–0.385], *p*(OLS) = 0.00), whereas no significant associations were observed with BF or grip strength (all standardized β < 0.2) (Table 4 and Figure 5). In the sensitivity analyses, quantile regression did not show significant associations with BMI or SMI (BMI *p*(QR) = 0.21; SMI *p*(QR) = 0.19), whereas the GAM demonstrated significant nonlinear effects on both indices (BMI *p*(GAM) = 0.01; SMI *p*(GAM) = 0.01) (Table 4 and Figure 6). After additional adjustment for total energy intake, sugar-sweetened beverage intake remained positively associated with BMI and SMI (BMI: β = 0.348 [0.117–0.580], *p*(OLS) = 0.00; SMI: β = 0.207 [0.043–0.370], *p*(OLS) = 0.02), and the GAM again confirmed significant nonlinear associations (BMI: *p*(GAM) = 0.00; SMI: *p*(GAM) = 0.02), whereas the results of quantile regression remained nonsignificant (BMI: *p*(QR) = 0.23; SMI: *p*(QR) = 0.11) (Table 4 and Figure 6). A similar zero-inflated pattern in sugar-sweetened beverage intake may partially account for the divergence between OLS/GAM (significant) and quantile regression (nonsignificant). No significant associations with BF or grip strength were observed in any model.

Milk-containing beverages were not associated with BMI, BF, or grip strength in any model (all standardized β < 0.2) (Table 4 and Figure 5). However, consistent positive associations were observed with the SMI. In the age- and sex-adjusted model, milk intake was positively associated with the SMI (β = 0.209 [0.055–0.363], *p*(OLS) = 0.01) (Table 4). In the sensitivity analyses, quantile regression did not reach significance (*p*(QR) = 0.27), whereas the GAM indicated a significant nonlinear association (*p*(GAM) = 0.01) (Table 4 and Figure 5). In the age-, sex-, and energy-adjusted model, the positive association between milk intake and the SMI persisted (β = 0.183 [0.020–0.346], *p*(OLS) = 0.03); quantile regression again was not significant (*p*(QR) = 0.39), but the GAM continued to have a significant effect (*p*(GAM) = 0.03) (Table 4 and Figure 5). This discrepancy among the statistical models is also consistent with the zero-inflated pattern of milk beverage consumption, where many participants reported no intake and only a subset consumed meaningful amounts. This distribution amplifies the effects detected by OLS and nonlinear GAMs while attenuating median-based effects in quantile regression.

Alcohol intake was not significantly associated with BMI, BF, SMI, or grip strength in either the age- or sex-adjusted model or the age-, sex-, or energy-adjusted model, with all standardized β values less than 0.2. Sensitivity analyses via quantile regression and the GAM likewise did not detect any significant associations for these outcomes.

In summary, the consumption of sugar-sweetened beverages was consistently positively associated with BMI and the SMI, irrespective of adjustment for total energy intake. Milk-containing beverages were associated exclusively with the SMI, and this association remained evident after additional adjustment for total energy and in nonlinear models. Total beverage intake showed partial associations with BMI and the SMI, particularly for the SMI, but these associations were less consistent than those observed for sugar-sweetened beverages. Alcohol intake was not associated with any body composition index. In terms of nutrient intake, milk and sugar-sweetened beverages were positively associated with energy and carbohydrates in the age- and sex-adjusted models, but these associations generally disappeared after adjusting for total energy intake, suggesting that their effects may be at least partially mediated by increased overall energy consumption.

## 4. Discussion

In this study, we comprehensively examined the associations between beverage-derived energy intake, body composition, and nutrient intake among Japanese adults aged 20–39 years. We found that total beverage-derived energy was strongly correlated with energy from sugar-sweetened beverages, suggesting that a substantial proportion of beverage-derived energy originates from sugary drinks. In the age- and sex-adjusted models, total beverage-derived energy and sugar-sweetened beverage-derived energy were positively associated with total energy intake and carbohydrate intake. Milk-based beverage intake was also positively associated with total energy intake and the intake of carbohydrates, fat, and protein; however, these associations disappeared after additional adjustment for total energy intake. These findings indicate that the beverage-derived caloric content largely reflects overall dietary energy intake.

In contrast, the associations of beverage-derived energy with BMI and the SMI persisted even after further adjustment for total energy intake, suggesting a potential contribution of beverage-derived calories to body composition. Generalized additive models revealed a linear pattern of associations between total beverage-derived energy and sugar-sweetened beverage-derived energy with both BMI and SMI, whereas milk-based beverages displayed a nonlinear pattern characterized by a plateau in the SMI at higher intake levels. Taken together, these findings suggest that, particularly among younger adults in their 20 s and 30 s, higher consumption of sugar-sweetened beverages may be associated with greater body weight, whereas modest intake of milk-based beverages may be associated with a higher SMI without concurrent increases in BMI or body fat percentage in this study.

Given that beverage intake was highly skewed, with a substantial proportion of participants reporting no intake, the distribution became zero-inflated. In such cases, OLS and GAMs are likely to detect differences driven by the upper end of the intake distribution, whereas quantile regression, which focuses on the median, may fail to identify an effect. This imbalanced distribution may explain why OLS and GAMs showed significant associations, whereas quantile regression did not. In fact, both SMI and SSB showed a curved relationship, and the results were influenced by high consumers; thus, even though OLS and GAM showed significant differences, no significant differences were observed with QR. On the basis of the results of this preliminary examination, it will be necessary in the future to set an appropriate statistical sample size and to consider methodologically more suitable two-part models or zero-inflated modeling approaches for distributions with many zeros.

In this study, a model adjusted only for age and sex showed that SSB intake was positively associated with BMI, total energy intake, and carbohydrate intake. When total energy intake was additionally included as a covariate, only the positive association between SSB intake and BMI remained, whereas the associations with total energy and carbohydrate intake disappeared. In both observational studies and intervention trials, sugar-sweetened beverages have consistently been reported to be associated with greater increases in body weight and BMI as intake increases [6,7,8,18]. Moreover, the positive association between SSB intake and BMI even after adjustment for total energy intake in the present study suggests that SSB intake may affect BMI not only through increased energy intake but also via energy-independent pathways, such as altered appetite regulation and metabolic disturbances, which is consistent with findings from previous reviews and meta-analyses [6,7,8,18]. To date, the relationship between obesity and SSB consumption has been reported mainly in Western populations [6,7,8,18], and epidemiological data in Japanese populations could not be found [19]. Few studies from Japan have examined the association between added sugar intake and cardiovascular risk [19]. In the analysis of the 2016 National Health and Nutrition Survey in Japan, free sugar intake was inversely associated with blood pressure (men only) and HDL-C level (both sexes) and positively associated with total cholesterol level (women only) and LDL-C level (both sexes), whereas no associations were observed with BMI, WC, or HbA1c level. Thus, our findings provide valuable evidence to fill this gap. Therefore, this study suggested that reducing sugar intake from SSBs may represent an important intervention target to prevent excessive energy intake and subsequent obesity. To discuss the relationship between soft drink consumption and body composition in Japan, it is worthwhile to conduct surveys in the future on larger groups of Japanese people encompassing a wider age range.

The SMI increased with low to moderate intake of sugar-sweetened beverages but decreased with high consumption. In a cross-sectional study examining skeletal muscle mass index (SMI), dietary habits, and physical activity among Japanese university students, the SMI was significantly associated with energy intake in women [20]. A cross-sectional study of adolescents in China revealed that groups with a higher frequency of consumption of sugar-sweetened beverages had lower levels of muscle strength (such as grip strength). Logistic regression analysis revealed that, overall, Chinese adolescents who consumed sugary drinks 1–2 times/week (OR = 1.207, 95% CI: 1.132–1.287) and ≥3 times/week (OR = 1.771, 95% CI: 1.611–1.947) had lower muscle strength than those who consumed sugary drinks <1 time/week (*p* < 0.01) [21]. This result may suggest that SSBs are consumed in greater amounts by individuals with high energy demands and activity levels but that excessive intake may negatively impact muscle mass through high sugar consumption and increased fat accumulation. One hypothesis for the positive association between SSB intake and the SMI is that adequate carbohydrate intake may contribute to the maintenance and increase in the SMI by suppressing the use of muscle protein for energy (the protein-sparing effect) [22]. In other age groups and populations with different backgrounds, higher consumption of sugar-sweetened beverages has been associated with lower muscle mass or reduced muscle strength [21,23,24], suggesting that the positive associations observed in our young adult sample may not be generalizable to all populations.

Next, milk-based beverages were also examined. After adjusting for age and sex, low to moderate milk consumption increased protein and fat intake but actually decreased carbohydrate content. This is because, regardless of energy adjustment, a curvilinear effect was observed between milk consumption and protein intake, and it is possible that the results of some high-volume consumers influenced the lack of significance in the QR analysis. Moreover, when the main ingredient is milk, an increase in energy intake is accompanied by increased protein and fat intake, making it highly caloric. After adjusting for energy, only a relationship with protein intake was observed. The WHO and various national nutritional guidelines also recommend reducing beverages containing free sugars (such as SSBs and sweetened milk drinks) and instead choosing water, unsweetened beverages, and nutrient-rich beverages (such as low-fat milk) [25]. Our findings suggest that the consumption of an appropriate amount of milk may be useful for protein supplementation to maintain muscle function in young individuals.

There is debate regarding the interpretation of approaches that adjust for total energy intake, depending on whether it serves as a confounding factor or a mediating variable [26,27]. In this study, we judged it appropriate to adjust for total energy intake as a confounding factor for the following reasons. This study did not control for unmeasured confounders (such as physical activity, body size, and basal metabolic rate—important confounding factors), so total energy intake functions as a proxy for these variables [26]. Evaluation of compositional contribution: Energy derived from SSB accounts for approximately 5% of total energy intake, which is considerably lower than the 20–30% threshold at which prior studies suggest that mediating properties become prominent [27]. Therefore, with this adjustment, the effect of SSB can be interpreted as “the effect under equal total energy intake,” providing a useful estimate in the context of public health interventions. However, since total energy intake is not a perfect proxy, the possibility of residual confounding cannot be ruled out.

This study has several limitations. First, this was an exploratory study, and no a priori sample size calculation was performed. The number of participants in this study was limited to 76. Neither an a priori sample size calculation nor a formal power analysis was conducted; instead, all available participants (N = 76) were included as a convenience sample. Although the number of covariates entered into the models was restricted to a maximum of four (age, sex, total energy intake, and the main independent variable), which is broadly compatible with common rules of thumb such as approximately 10 observations per parameter, the study may still have been underpowered to detect small effect sizes. Second, in the analysis of potential nonlinear associations via generalized additive models (GAMs), the relatively modest sample size and the limited number of individuals with high SSB intake reduce the ability to detect and precisely estimate complex nonlinear patterns. Therefore, the GAM analyses in this study should be regarded as exploratory and intended mainly to assess whether there was strong evidence against the linearity assumption, and the detailed shape of the fitted smooth terms should be interpreted with caution. Moreover, nonsignificant findings in sensitivity analyses (e.g., quantile regression) may reflect insufficient statistical power (Type II error) rather than a true absence of associations, particularly given the high proportion of zero values.

Second, the participants were employees of a medical university, a group likely to have relatively high literacy regarding diet; therefore, caution is needed when generalizing these findings to the general Japanese population. To assess the validity of these results in relation to the entire Japanese population, it is necessary to conduct a new study with a larger sample size and a broader age range. Third, because of the cross-sectional design, causal relationships cannot be inferred from the observed associations. Fourth, dietary intake was assessed via self-administered questionnaires and recall difficulties, or social desirability may have led to underreporting, including total energy intake. Although total energy intake and beverage-derived energy were used for within-person comparisons, which may partly mitigate this bias, they cannot be completely ruled out.

Fifth, some potential confounders, such as physical activity, sleep, income, and educational attainment, were not fully assessed, and residual confounding may remain. Therefore, we chose a parsimonious adjustment set focusing on age, sex, BMI, and total energy intake. Adjustment for total energy intake is a standard approach in nutritional epidemiology to partly account for differences in body size, physical activity, and metabolic efficiency that are otherwise difficult to measure accurately. Nonetheless, we acknowledge that residual confounding by unmeasured or imprecisely measured factors, including physical activity, sleep, and socioeconomic status, may remain, especially in analyses of SMI and grip strength.

Our study population consisted of health professionals who are mostly highly educated (with a college degree or higher) and are likely to have relatively high income levels, which may confer some degree of homogeneity. In a systematic review on health literacy and SSB intake, three studies reported that individuals with higher health literacy had lower SSB intake [28]. According to one study, individuals with higher literacy tend to have better overall diet quality and lower SSB consumption [29]. On the other hand, reports suggest that health literacy is not strongly related and that only age, sex, and subnational level are associated, indicating inconsistent findings [30]. As seen from these results, in addition to health literacy, various factors, such as age (younger age), gender (male), and knowledge about where one lives are involved in SSB consumption, cannot necessarily be said that these results reflect the situation across Japan as a whole. However, considering that such research reports have not yet been published in Japan, this is thought to be significant.

The categories of SSB and dairy beverages did not fully capture the diversity of commercially available products, and we were unable to distinguish specific product characteristics, such as the presence of noncaloric sweeteners or the fat content. Although additional analyses were conducted using the frequency of non-caloric sweetener intake as an exposure, no statistically significant associations were observed with the outcomes in this study.

The BEVQ-15 is a simple self-report tool, but there are several limitations to the BEVQ-15 used in this study. The BEVQ-15 is a self-administered questionnaire and may be subject to recall bias and social desirability bias [12,31]. In addition, since it consolidates beverages into 15 categories, it may underestimate the intake of specific beverages or fail to adequately capture the consumption levels of individuals with extremely high intake [32]. Furthermore, the questionnaire was developed and validated primarily for use with adult populations in the United States, so further translation, cultural adaptation, and validation are necessary for generalization to other cultures and age groups [33].

Finally, our beverage intake data exhibited a zero-heavy and right-skewed distribution, for which specialized approaches such as two-part or zero-inflated models could be considered more appropriate. In this study, however, our primary aim was to characterize average differences and covariate associations in intake, and we therefore chose to rely on OLS, quantile regression, and GAMs as our main analytic approaches, balancing model complexity against the available sample size. Consequently, we did not explicitly model the potentially distinct processes underlying any consumption versus the amount consumed among drinkers, which represents a methodological limitation of our work and should be addressed in future studies using two-part or zero-inflated modeling frameworks.

## 5. Conclusions

In conclusion, we found that among employees at a Japanese university, higher intake of sugar-sweetened beverages was positively associated with BMI, whereas milk intake was associated with the SMI. Future research should clarify the mechanisms through which sugar-sweetened beverages may increase BMI beyond their contribution to total energy intake, for example, by examining differences in blood glucose and appetite-related hormone levels, as well as eating behaviors such as meal duration and eating speed, between consumers and non-consumers. In addition, because evidence in Japanese populations remains limited, large-scale epidemiological studies that incorporate economic and social background factors are warranted to further verify and extend the present findings.

## Figures and Tables

**Table 1:** Baseline characteristics of the participants.

Variable	Male (Mean ± SD), *n* = 35	Female (Mean ± SD), *n* = 41	*p* Value	Effect Size
Age *	29.97 ± 4.67	27.29 ± 4.53	0.011	Cliff’s delta = 0.34 (95%CI 0.068–0.59)
BMI	22.66 ± 2.78	21.56 ± 2.69	0.084	Cohen’s d = 0.404 (95%CI −0.052–0.86)
%Body Fat	21.54 ± 5.99	30.69 ± 6.07	0.00	Cohen’s d = −1.517 (95%CI −2.03–−1.004)
SMI	9.9 ± 0.96	8.01 ± 0.88	0.00	Cohen’s d = 2.056 (95%CI 1.496–2.616)
Grip strength	38.15 ± 7.92	24.15 ± 5.68	0.00	Cohen’s d = 2.059 (95%CI 1.499–2.619)
Energy *	1898.0 ± 539.68	1422.15 ± 431.29	0.00	Cliff’s delta = 0.54 (95%CI 0.306–0.744)
Protein	66.74 ± 18.86	49.8 ± 14.68	0.00	Cohen’s d = 1.013 (95%CI 0.533–1.492)
Carbohydrate *	271.05 ± 100.3	198.54 ± 71.52	0.00	Cliff’s delta = 0.477 (95%CI 0.247–0.695)
Fat *	53.75 ± 17.89	42.6 ± 12.97	0.0058	Cliff’s delta = 0.37 (95%CI 0.112–0.61)
Drink (Total) *	167.77 ± 235.53	106.8 ± 123.67	0.34	Cliff’s delta = 0.128 (95%CI −0.134–0.392)
Drink (Alc) *	35.16 ± 154.55	13.42 ± 36.86	0.53	Cliff’s delta = −0.063 (95%CI −0.252–0.136)
Drink (Milk) *	53.17 ± 130.03	31.91 ± 43.41	0.67	Cliff’s delta = −0.055 (95%CI −0.31–0.194)
Drink (Sugar) *	69.91 ± 100.12	53.38 ± 102.17	0.15	Cliff’s delta = 0.19 (95%CI −0.071–0.437)

Group differences were examined via the independent samples *t* test or the Mann–Whitney U test (*, as appropriate). Effect sizes were calculated as Cohen’s d for *t* tests and Cliff’s delta for Mann–Whitney U tests. Drink (Total), Drink (Alc), Drink (Milk), and Drink (Sugar) represent daily intake (energy).

**Table 2:** The correlations between beverage-derived energy and total beverage intake (drink (total)), sugar-sweetened beverages, milk-containing beverages, or alcoholic beverages.

var1	var2	N	rho_ci_*p*
Drink (Alc)	Drink (Milk)	76	−0.21 (95%CI −0.40 to −0.02), *p* = 0.063
Drink (Alc)	Drink (Sugar)	76	0.06 (95%CI −0.16 to 0.28), *p* = 0.59
Drink (Alc)	Drink(total)	76	0.28 (95%CI 0.08 to 0.49), *p* = 0.013
Drink (Milk)	Drink (Sugar)	76	0.19 (95%CI −0.04 to 0.42), *p* = 0.097
Drink (Milk)	Drink(total)	76	0.50 (95%CI 0.29 to 0.68), *p* = 0.000
Drink (Sugar)	Drink(total)	76	0.74 (95%CI 0.57 to 0.86), *p* = 0.000

The correlation between each drink type was estimated by Spearman’s rho. The data are presented as rho values with 95% CIs and *p* values. Red letters indicate significance (*p* < 0.05).

**Table 3:** Associations of beverage intake with energy and nutrient intake adjusted with Model 1 (age + sex) and Model 2 (age + sex + energy).

Outcome	Predictor	Age + Sex	Age + Sex + Energy
**Energy**	Drink (total)	0.263 [0.064 to 0.462], *p*(OLS) = 0.01, *p*(QR) = 0.01, *p*(GAM) = 0.01	NA
**Carbohydrate**	Drink (total)	0.252 [0.045 to 0.458], *p*(OLS) = 0.02, *p*(QR) = 0.01, *p*(GAM) = 0.02	−0.001 [−0.085 to 0.084], *p*(OLS) = 0.99, *p*(QR) = 0.27, *p*(GAM) = 0.53
**Fat**	Drink (total)	0.126 [−0.090 to 0.342], *p*(OLS) = 0.26, *p*(QR) = 0.29, *p*(GAM) = 0.37	−0.089 [−0.239 to 0.061], *p*(OLS) = 0.25, *p*(QR) ≤ 0.001, *p*(GAM) = 0.25
**Protein**	Drink (total)	0.113 [−0.094 to 0.320], *p*(OLS) = 0.29, *p*(QR) = 0.53, *p*(GAM) = 0.29	−0.079 [−0.234 to 0.077], *p*(OLS) = 0.32, *p*(QR) = 0.06, *p*(GAM) = 0.45
**Energy**	Drink (Sugar)	0.289 [0.092 to 0.487], *p*(OLS) = 0.01, *p*(QR) = 0.00, *p*(GAM) = 0.01	NA
**Carbohydrate**	Drink (Sugar)	0.336 [0.135 to 0.536], *p*(OLS) = 0.00, *p*(QR) = 0.01, *p*(GAM) = 0.00	0.065 [−0.019 to 0.149], *p*(OLS) = 0.13, *p*(QR) = 0.12, *p*(GAM) = 0.13
**Fat**	Drink (Sugar)	0.124 [−0.093 to 0.340], *p*(OLS) = 0.27, *p*(QR) = 0.08, *p*(GAM) = 0.27	−0.116 [−0.267 to 0.034], *p*(OLS) = 0.13, *p*(QR) = 0.20, *p*(GAM) = 0.19
**Protein**	Drink (Sugar)	0.133 [−0.074 to 0.339], *p*(OLS) = 0.21, *p*(QR) = 0.17, *p*(GAM) = 0.21	−0.079 [−0.236 to 0.078], *p*(OLS) = 0.33, *p*(QR) = 0.85, *p*(GAM) = 0.41
**Energy**	Drink (Milk)	0.283 [0.087 to 0.479], *p*(OLS) = 0.01, *p*(QR) = 0.33, *p*(GAM) = 0.01	NA
**Carbohydrate**	Drink (Milk)	0.265 [0.061 to 0.469], *p*(OLS) = 0.01, *p*(QR) = 0.00, *p*(GAM) = 0.01	−0.007 [−0.091 to 0.078], *p*(OLS) = 0.88, *p*(QR) = 0.32, *p*(GAM) = 0.00
**Fat**	Drink (Milk)	0.235 [0.025 to 0.444], *p*(OLS) = 0.03, *p*(QR) = 0.53, *p*(GAM) = 0.05	0.013 [−0.139 to 0.164], *p*(OLS) = 0.87, *p*(QR) = 0.11, *p*(GAM) = 0.00
**Protein**	Drink (Milk)	0.272 [0.075 to 0.469], *p*(OLS) = 0.01, *p*(QR) = 0.69, *p*(GAM) = 0.01	0.081 [−0.074 to 0.237], *p*(OLS) = 0.31, *p*(QR) = 0.72, *p*(GAM) = 0.00
**Energy**	Drink (Alc)	−0.081 [−0.285 to 0.124], *p*(OLS) = 0.44, *p*(QR) = 0.88, *p*(GAM) = 0.44	NA
**Carbohydrate**	Drink (Alc)	−0.130 [−0.340 to 0.079], *p*(OLS) = 0.23, *p*(QR) = 0.92, *p*(GAM) = 0.23	−0.053 [−0.132 to 0.026], *p*(OLS) = 0.19, *p*(QR) = 0.90, *p*(GAM) = 0.16
**Fat**	Drink (Alc)	−0.108 [−0.321 to 0.106], *p*(OLS) = 0.33, *p*(QR) = 0.81, *p*(GAM) = 0.43	−0.044 [−0.187 to 0.099], *p*(OLS) = 0.55, *p*(QR) = 0.91, *p*(GAM) = 0.55
**Protein**	Drink (Alc)	−0.169 [−0.371 to 0.033], *p*(OLS) = 0.11, *p*(QR) = 0.80, *p*(GAM) = 0.11	−0.113 [−0.259 to 0.033], *p*(OLS) = 0.13, *p*(QR) = 0.88, *p*(GAM) = 0.13

The models used are linear regression (OLS), median quantile regression (τ = 0.5), and generalized additive models (GAMs). Beverage intake (Drink (Alc), Drink (milk), Drink (sugar), Drink (total)) was entered as the exposure variable, and nutrient intake (energy, carbohydrate, protein, fat) was treated as the outcome. All the models were adjusted for age and sex (Model 1) and for total energy intake (Model 2). Missing data were handled by complete-case analysis. Standardized regression coefficients (β) and 95% confidence intervals are presented for OLS, whereas quantile regression uses the nid method to estimate standard errors. GAMs with thin plate regression splines for beverage intake were fitted via the mgcv package, and nonlinear associations were considered statistically significant when *p* < 0.05 and effective degrees of freedom ≥ 2. Red letters indicate significance (*p*(OLS) < 0.05).

**Table 4:** Associations of beverage intake with body size (BMI, BF, SMI) and grip strength adjusted with Model 1 (age + sex) and Model 2 (age + sex + energy).

Outcome	Predictor	Age + Sex	Age + Sex + Energy
**BMI**	Drink(total)	0.233 [0.008 to 0.457], *p*(OLS) = 0.05, *p*(QR) = 0.71, *p*(GAM) = 0.09	0.253 [0.018 to 0.489], *p*(OLS) = 0.04, *p*(QR) = 0.71, *p*(GAM) = 0.08
**BF**	Drink(total)	−0.025 [−0.211 to 0.161], *p*(OLS) = 0.79, *p*(QR) = 0.80, *p*(GAM) = 0.79	0.012 [−0.182 to 0.206], *p*(OLS) = 0.90, *p*(QR) = 0.45, *p*(GAM) = 0.91
**SMI**	Drink(total)	0.263 [0.112 to 0.415], *p*(OLS) = 0.00, *p*(QR) = 0.04, *p*(GAM) = 0.00	0.243 [0.085 to 0.402], *p*(OLS) = 0.00, *p*(QR) = 0.03, *p*(GAM) = 0.00
**Grip strength**	Drink(total)	0.032 [−0.131 to 0.195], *p*(OLS) = 0.70, *p*(QR) = 0.72, *p*(GAM) = 0.70	0.028 [−0.143 to 0.200], *p*(OLS) = 0.75, *p*(QR) = 0.80, *p*(GAM) = 0.75
**BMI**	Drink (Sugar)	0.314 [0.094 to 0.533], *p*(OLS) = 0.01, *p*(QR) = 0.21, *p*(GAM) = 0.01	0.348 [0.117 to 0.580], *p*(OLS) = 0.00, *p*(QR) = 0.23, *p*(GAM) = 0.00
**BF**	Drink (Sugar)	0.057 [−0.130 to 0.243], *p*(OLS) = 0.55, *p*(QR) = 0.72, *p*(GAM) = 0.42	0.107 [−0.087 to 0.302], *p*(OLS) = 0.28, *p*(QR) = 0.80, *p*(GAM) = 0.30
**SMI**	Drink (Sugar)	0.231 [0.076 to 0.385], *p*(OLS) = 0.00, *p*(QR) = 0.19, *p*(GAM) = 0.01	0.207 [0.043 to 0.370], *p*(OLS) = 0.02, *p*(QR) = 0.11, *p*(GAM) = 0.02
**Grip strength**	Drink (Sugar)	0.017 [−0.146 to 0.180], *p*(OLS) = 0.84, *p*(QR) = 0.67, *p*(GAM) = 0.84	0.011 [−0.162 to 0.185], *p*(OLS) = 0.90, *p*(QR) = 1.00, *p*(GAM) = 0.90
**BMI**	Drink (Milk)	0.093 [−0.134 to 0.321], *p*(OLS) = 0.42, *p*(QR) = 0.63, *p*(GAM) = 0.42	0.103 [−0.139 to 0.344], *p*(OLS) = 0.41, *p*(QR) = 0.54, *p*(GAM) = 0.41
**BF**	Drink (Milk)	−0.088 [−0.272 to 0.095], *p*(OLS) = 0.35, *p*(QR) = 0.69, *p*(GAM) = 0.35	−0.055 [−0.249 to 0.138], *p*(OLS) = 0.58, *p*(QR) = 0.76, *p*(GAM) = 0.66
**SMI**	Drink (Milk)	0.209 [0.055 to 0.363], *p*(OLS) = 0.01, *p*(QR) = 0.27, *p*(GAM) = 0.01	0.183 [0.020 to 0.346], *p*(OLS) = 0.03, *p*(QR) = 0.39, *p*(GAM) = 0.03
**Grip strength**	Drink (Milk)	0.127 [−0.032 to 0.286], *p*(OLS) = 0.12, *p*(QR) = 0.68, *p*(GAM) = 0.12	0.134 [−0.035 to 0.302], *p*(OLS) = 0.12, *p*(QR) = 0.65, *p*(GAM) = 0.12
**BMI**	Drink (Alc)	0.005 [−0.223 to 0.233], *p*(OLS) = 0.97, *p*(QR) = 1.00, *p*(GAM) = 0.97	0.005 [−0.225 to 0.236], *p*(OLS) = 0.96, *p*(QR) = 1.00, *p*(GAM) = 0.96
**BF**	Drink (Alc)	−0.008 [−0.192 to 0.176], *p*(OLS) = 0.93, *p*(QR) = 0.99, *p*(GAM) = 0.94	−0.019 [−0.203 to 0.165], *p*(OLS) = 0.84, *p*(QR) = 0.95, *p*(GAM) = 0.84
**SMI**	Drink (Alc)	0.022 [−0.139 to 0.183], *p*(OLS) = 0.79, *p*(QR) = 0.98, *p*(GAM) = 0.79	0.035 [−0.125 to 0.194], *p*(OLS) = 0.67, *p*(QR) = 0.30, *p*(GAM) = 0.67
**Grip strength**	Drink (Alc)	−0.103 [−0.262 to 0.056], *p*(OLS) = 0.21, *p*(QR) = 0.00, *p*(GAM) = 0.21	−0.102 [−0.263 to 0.059], *p*(OLS) = 0.22, *p*(QR) = 0.84, *p*(GAM) = 0.22

The models used are linear regression (OLS), median quantile regression (τ = 0.5), and generalized additive models (GAMs). Beverage intake (Drink (Alc), Drink (milk), Drink (sugar), Drink (total)) was entered as an exposure variable, and body size indices (BMI, SMI, BF, grip strength) were treated as outcomes. All the models were adjusted for age and sex (Model 1) and for total energy intake (Model 2). Missing data were handled by complete-case analysis. Standardized regression coefficients (β) and 95% confidence intervals are presented for OLS, whereas quantile regression uses the nid method to estimate standard errors. GAMs with thin plate regression splines for beverage intake were fitted via the mgcv package, and nonlinear associations were considered statistically significant when *p* < 0.05 and effective degrees of freedom ≥ 2. Red letters indicate significance (*p*(OLS) < 0.05).

## Data Availability

Some or all datasets generated during and/or analyzed during the current study are not publicly available due to further analysis but are available from the corresponding author upon reasonable request.

## Abbreviations

The following abbreviations are used in this manuscript:

BDHQ	Brief-Type Self-Administered Diet History Questionnaire
BEVQ-15	Beverage Intake Questionnaire-15
OLS	Ordinary least squares
QR	Quantile regression
GAM	Generalized Additive Model
SSB	Sugar-sweetened beverage
